# ChikDenMaZika Syndrome: the challenge of diagnosing arboviral infections in the midst of concurrent epidemics

**DOI:** 10.1186/s12941-016-0157-x

**Published:** 2016-07-22

**Authors:** Alberto E. Paniz-Mondolfi, Alfonso J. Rodriguez-Morales, Gabriela Blohm, Marilianna Marquez, Wilmer E. Villamil-Gomez

**Affiliations:** Department of Pathology and Laboratory Medicine, Hospital Internacional, Barquisimeto, Venezuela; Laboratory of Biochemistry, Instituto de Biomedicina/IVSS, Caracas, Venezuela; Colombian Collaborative Network on Zika (RECOLZIKA), Pereira, Risaralda Colombia; Public Health and Infection Research Group, Faculty of Health Sciences, Universidad Tecnologica de Pereira, Pereira, Risaralda Colombia; Organización Latinoamericana Para el Fomento de la Investigación en Salud (OLFIS), Bucaramanga, Santander Colombia; Committee on Zoonoses and Haemorrhagic Fevers, Asociación Colombiana de Infectología (ACIN), Bogotá, DC Colombia; Department of Biology, University of Florida, Gainesville, FL USA; Infectious Diseases and Infection Control Research Group, Hospital Universitario de Sincelejo, Sincelejo, Sucre Colombia; Programa del Doctorado de Medicina Tropical, Universidad del Atlántico, Barranquilla, Atlántico, Colombia

Arthropod-borne viruses are becoming and increasing threat worldwide, especially in the New World, which has recently witnessed an unprecedented outburst of Arboviral outbreaks [1–4], such as the recent and ongoing chikungunya (CHIKV) [1] and Zika (ZIKV) [2] epidemics throughout the Pacific and the Americas. These emerging viral infections are largely due to a number of factors such as climate change [5–7], ever-increasing trends towards urbanization and growing travel and commercial exchange activities [8–12]; which have led to a spillover of these pathogens from their naturally occurring sylvatic niches and reservoirs into susceptible urban settings and newly unexposed geographic areas [13–16].

Other arbovirus such as yellow fever (YFV) and dengue (DENV) [6, 17], have been circulating for longer in the American continent exhibiting endo-epidemic cycles influenced by a number of enabling drivers such as poor socioeconomic conditions, climate variations, migration from rural to urban locations, among others [3, 4, 6, 7, 11–13, 17]. The complex but at the same time suitable eco-epidemiological conditions of tropical and subtropical regions has privileged the persistence of arboviruses by providing an ideal blend in terms of vector usage and specificity as well as an ample host range. Actually, newly introduced arboviruses in the New World seem to be smoothly transitioning from their epidemic effervescence into endemic levels of transmission revealing an increased potential for adaptation [1–3, 16].

Arboviruses in Latin America include a number of pathogens belonging to different families such as the Flaviviridae (DENV and ZIKV) [3, 18], Togaviridae (CHIKV and MAYV) [16, 19–21], and Bunyaviridae (Oropouche virus, OROV) [22] just to name a few; with their biologic behavior and pathogenicity exhibiting distinct features but also great similarities. In particular, for ZIKV, the fact that this virus along with its recent introduction has also found a naïve population with no pre-existing endemic cycles in the region, appears to have pressed for the appearance of new variants with an increased pathogenic potential such as recently observed in the evolving epidemic with a disproportionate number of atypical clinical manifestations and complications not seen before elsewhere [2, 9, 23, 24].

Although 80 % of cases are asymptomatic, symptoms of ZIKV classically include mild or no fever, pruriginous maculopapular rash, conjunctivitis, arthralgia and myalgia, headache, malaise and fatigue (Table 1) [3, 25]. Notably, most of these symptoms can easily be confused with those of CHIKV, MAYV and DENV infections with a high chance of misdiagnosing such cases especially during early clinical stages, thus posing a significant diagnostic challenge amongst these arboviral-like illnesses (Table 1) [3, 25]. Differentiation on clinical grounds alone is often a very difficult task and requires a high degree of experience and clinical insight, despite the occurrence of distinct signs and symptoms such as focal joint edema of distal aspects of the limbs in ZIKV, meningism in OROV and retro/orbital pain and hemorrhagic diathesis in DENV (Table 1) [3, 25].

**Table 1:** Main clinical findings in the ChikDenMaZika Syndrome [3, 25]

Clinical findings	ChikDenMaZika Arboviruses			
	CHIK	DEN	MA	ZIKA
Fever	+++	*++++*	++++	++/0^a^
Myalgia/arthralgia	*++++*	+++	+++	++
Edema in limbs	0	0	0	*++*
Maculopapular rash	++	++	++	*+++* ^b^
Retro-ocular pain	+	*++*	++	++
Conjunctivitis, non-purulent	+	0	0	*+++*
Lymphadenopathies	++	*++*	+	+
Hepatomegaly	*++*	0	+	0
Leukopenia/thrombocytopenia	++	*+++*	++	0/+^c^
Hemorrhages	+	*+++*	0	0/+^c^

Italics indicates for which of the arboviruses is the highest frequency of the clinical finding

^a^Depends on geography and phylogeny of the virus, in some areas patients do not present fever

^b^Pruriginous (mild to severe)

^c^In some cases these findings have been reported [2]

Moreover, clinical findings and differentiation among arboviral infections becomes a more complicated task when dealing with special populations such as pregnant women and children, due to their tendency to develop perinatal complications, particularly in CHIKV and ZIKV infections [26, 27].

In addition, cocirculation and coinfection with different arboviruses is becoming a common phenomenon with cases reported from Africa (Nigeria and Angola, where an epidemic of YFV is ongoing, with imported cases to other continents) [28–30], the Caribbean (Haiti) [31], South America (Brazil and Colombia) [18, 32], and the Pacific (New Caledonia) [33]. Also, cases of coinfection with other endemic pathogens such as malaria (still a public health threat in areas of South East Asia and Latin America) [28], as well as other viral illnesses such as HIV are being increasingly reported [34, 35]. The broad range of possible coinfecting agents and the non-specific signs and symptoms at the initial stages of infection complicate even more the diagnostic approach to these cases, beyond the clinical aspects, implying as well the needs for the so called multiplex diagnostic tools [36].

Interestingly, confounding diagnosis between pathogens exhibiting similar clinical features and common geographical and epidemiological grounds, is not an uncommon scenario. For example, in several Asian countries were hepatorenal syndrome causing-hantavirus is endemic, there is usually a significant overlap of symptoms with cases of leptospirosis and infection by the scrub-typhus causative agent *Orientia tsutsugamushi*. Given the similar clinical features and the variable but notable immune-reactivity to these agents revealed by seroepidemiological studies, the term “Lepthangamushi” was adopted to describe this overlapping clinical syndrome [37].

**Fig. 1:**
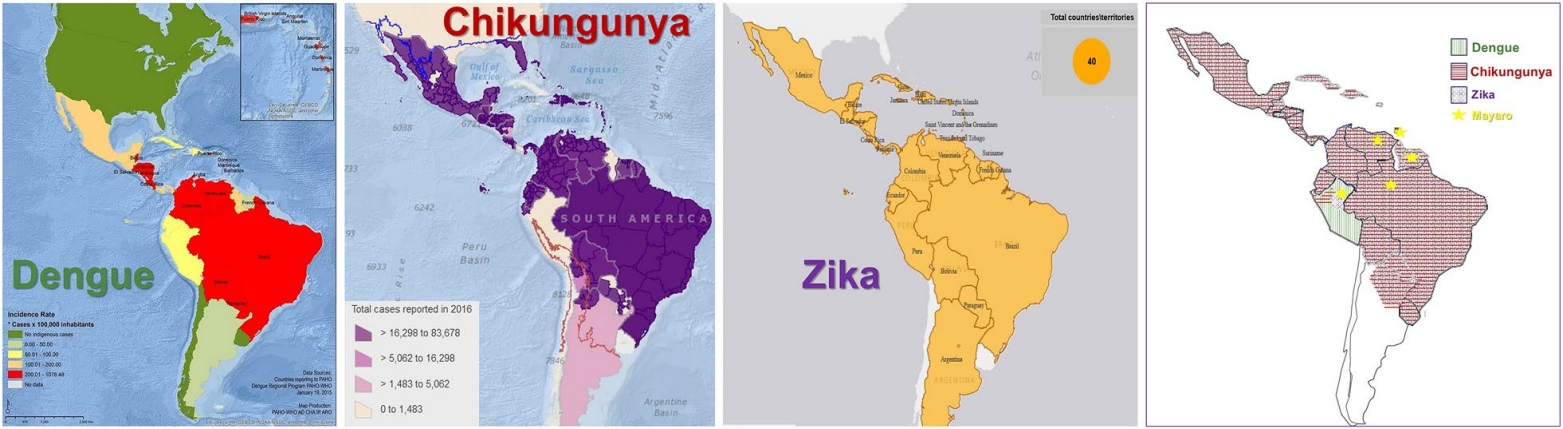
Reported distribution of DENV, CHIKV, ZIKV and MAYV in Latin America, based on PAHO and review of literature

As a result of our field work in South America [3, 4, 8–12], and in order to establish an effective clinical pattern recognition approach and diagnostic management strategy, in cases of infections suggestive of arthropod-borne viruses, we have coined the term “ChikDenMaZika syndrome” which groups the major causative players in our region (CHIKV, DENV, MAYV and ZIKV) (Fig. 1), as a mnemonic rule to include in our list of differentials at the time of diagnosis. Nevertheless, it is essential for clinicians to always keep in mind the other look-alike entities that commonly occur endemically in their areas of practice, such as other viral infections like parvovirus B19, enteroviral exanthems, adenovirus, rubella, measles; bacterial infections like rickettsiae, Q fever, leptospirosis, ehrlichiosis and *Streptococcus*; as well as parasitic disease such as malaria and acute Chagas disease [38–43].

Efforts should focus on the necessity to contain the ongoing concurrent epidemics (Fig. 1) and to maintain strict and continued surveillance programs to monitor the spread of these viruses as well as the introduction of newly emergent pathogens [3, 13, 16]. In the field as well as in low-income and remote areas, clinicians should take into consideration the overlapping clinical features shared amongst these agents as well as the possibility of co-infection in their differential diagnosis. Hopefully the term “ChikDenMaZika syndrome” will provide clinicians with a useful mnemonic tool that would aid in narrowing-down diagnosis when faced with arboviral-like disease symptoms such as fever, maculopapular rash, arthralgias, myalgias and non-purulent conjunctivitis (or conjunctival hyperemia). Such multi-agent targeted approach in clinical diagnostics should also be extrapolated to the laboratory bench by improving the usage of multiplex RT-PCR diagnostic platforms for arboviruses in returning travelers [36], as well as residents of endemic areas, given the increasing reported frequency of co-circulation of multiple arboviruses and its emerging threat in tropical regions [44].
